# Constructing the bundle sheath towards enhanced photosynthesis

**DOI:** 10.1093/jxb/erz537

**Published:** 2020-02-19

**Authors:** Pallavi Singh, Gregory Reeves

**Affiliations:** University of Cambridge, Department of Plant Sciences, Downing Site, Cambridge, UK

**Keywords:** Activation tagging, bundle sheath cells, C_4_ photosynthesis, Kranz anatomy, NAC052, transcriptional regulation

## Abstract

This article comments on:

**van Rooijen R, Schulze S, Petzsch P, Westhoff P**. 2020. Targeted misexpression of *NAC052*, acting in H3K4 demethylation, alters leaf morphological and anatomical traits in *Arabidopsis thaliana*. Journal of Experimental Botany 71, 1434–1448.

**C_4_ photosynthesis is a carbon-concentrating mechanism that alleviates photorespiratory losses by elevating the concentration of CO_2_ around Rubisco. Extant genes evolved to form this CO_2_-concentrating mechanism through changes in cell-preferential enzyme activity and leaf morphology (termed Kranz anatomy). Relatively little is known about regulatory *trans*-factors that promoted these changes. Using an activation tagging screen in C_3_*Arabidopsis thaliana*, Rooijen *et al.* (2019) identified NAC052, a H3K4 demethylase, as a possible regulator of an increased number of bundle sheath cells and chloroplasts. This study helps to decipher the genetic basis behind the evolutionary neofunctionalization of the bundle sheath in C_3_ species, which lead to morphological changes to facilitate C_4_ photosynthesis.**

The enzyme Rubisco plays a central role in photosynthesis by capturing atmospheric carbon dioxide (CO_2_) in an organic form. This enzymatic step is the basis of C_3_ photosynthesis. However, Rubisco also reacts with oxygen (O_2_) to generate a toxic by-product, which must be salvaged by an energy-intensive process known as photorespiration (Portis and Parry, 2007). Many lineages of plants have evolved means to prevent photorespiration by increasing the relative concentration of CO_2_ around Rubisco. In plants, the C_4_ cycle is the most prevalent of these mechanisms which involves spatial separation of the reactions of photosynthesis. CO_2_ is initially fixed in mesophyll (M) cells as a four carbon (C_4_) intermediate, diffused deeper inside the leaf into bundle sheath (BS) cells where the C_4_ intermediate is decarboxylated for refixation by Rubisco (Hatch and Slack, 1966). Therefore, C_4_ photosynthesis requires BS cell-specific expression of Rubisco, but also several other enzymes that must be M or BS specific in order to operate the cycle. In conjunction, a unique cellular arrangement in most C_4_ leaves (termed Kranz anatomy) has evolved to facilitate this molecular CO_2_ pump (El-Sharkawy and Hesketh, 1965; Hatch, 1987). Compared with C_3_ plants, C_4_ Kranz anatomy generally comprises denser venation, increased BS cell size, number, and chloroplast content, a greater reliance on the BS for photosynthesis, fewer M cells, and more plasmodesmata connections between the M and BS (Box 1). As Kranz anatomy is a multifaceted trait, identifying its genetic determinants has been a bottleneck in C_4_ photosynthetic research.

Box 1. **Comparison of C_3_ and C_4_ leaf anatomy**

A schematic of transverse cross-sections of mature C_4_*Flaveria trinervia* and C_3_*Arabidopsis thaliana* leaves. Cell outlines: upper and lower epidermis (black), vasculature (grey), bundle sheath (green), and mesophyll (pink). The middle layer of mesophyll cells (pink) highlights the difference in cell number between veins in C_3_ and C_4_ species. The dark green - color in C_4_ plants represents higher photosynthetic capacity of BS cells.

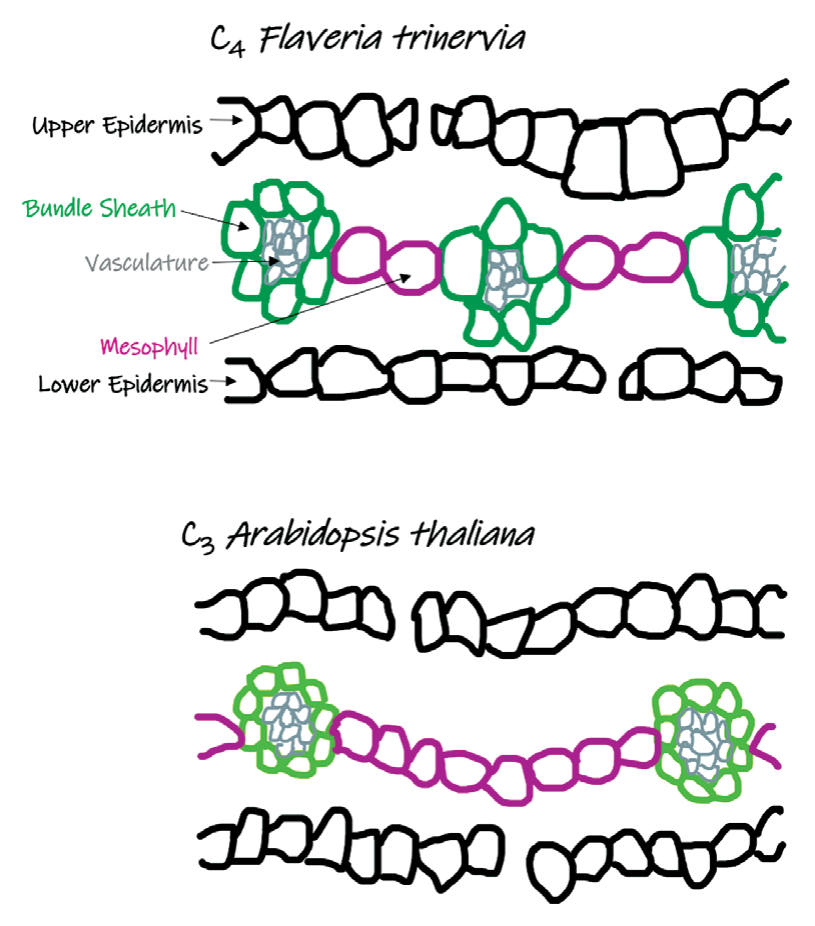

The transcription factors SCARECROW (SCR) and SHORTROOT (SHR) have been implicated with BS specification in C_4_ maize and C_3_ Arabidopsis (Slewinski *et al.*, 2012; Cui *et al.*, 2014). However, recent studies have shown that SCR/SHR regulate cell patterning in more broad contexts, such as root, epidermal, stomatal, and M cell patterning, suggesting that these two factors cannot fully account for the developmental changes in the BS to enable C_4_ photosynthesis (Hughes *et al.*, 2019). Paralogues of the maize GOLDEN2 (G2) transcription factor family regulate dimorphic chloroplast differentiation in BS and M cells (Wang *et al.*, 2013). Overexpression of GOLDEN2-LIKE1 in C_3_ rice led to increased chloroplast development in the vascular bundles of rice seedlings (Nakamura *et al.*, 2009). Thus, GOLDEN and GOLDEN-LIKE transcription factors play a role in plastid morphogenesis that probably aided in increasing the photosynthetic capacity of the BS. Beyond a few characterized regulatory steps that occur at multiple levels of gene expression, our current understanding of the steps required to engineer Kranz anatomy and cell-specific expression of C_4_ cycle enzymes is limited (Reeves *et al.*, 2017; Sedelnikova *et al.*, 2018).

## NAC052, a H3K4 demethylase: identified as a novel genetic regulator of bundle sheath anatomy in Arabidopsis

In this issue of the *Journal of Experimental Botany*, van Rooijen *et al.* (2019) used activation tagging to identify a regulator influencing the number and chloroplast content of BS cells in *A. thaliana* (Box 2). In activation tagging, a promoter is randomly inserted into a reference genome, which results in transcriptional changes of genes in close proximity to the insertion site (Tani *et al.*, 2004). In this study, the authors used an *A. thaliana* reference line from Döring *et al.* (2019) which was transformed with the promoter of the C_4_*Flaveria trinervia* GLYCINE DECARBOXYLASE P-SUBUNIT gene (pGLDPA_Ft_) to drive BS-preferential expression of a chloroplast-targeted green fluorescent protein (pGLDPA_Ft_::RbcS.TP-sGFP). In order to identify regulators that influence the morphology of the BS, they used a second BS-preferential promoter from the *F. trinervia* GLYCINE DECARBOXYLASE T-SUBUNIT gene (pGLDT_Ft_) as an activation tag. Altered GFP fluorescence relative to the reference line allowed screening to find individual lines with altered BS-related phenotypes. Genomic analysis of one such line revealed that the reference promoter had inserted in the coding sequence of the gene encoding NAC052, a transcriptional repressor involved in H3K4 demethylation (Ning *et al.*, 2015).

Box 2. **Activation tagging of NAC052 alters leaf anatomy and the expression of its target genes**

(A) Whole-plant morphology of transgenic lines transformed with various versions of NAC052, a H3K4 demethylase, into a reference background containing a bundle sheath-localized GFP signal. (B) Screening lines for differences in GFP intensity allowed detection of the enhanced number of chloroplasts in the bundle sheath and overall number of bundle sheath cells. (C) Comparative transcriptomics identified putative regulatory targets of NAC052.

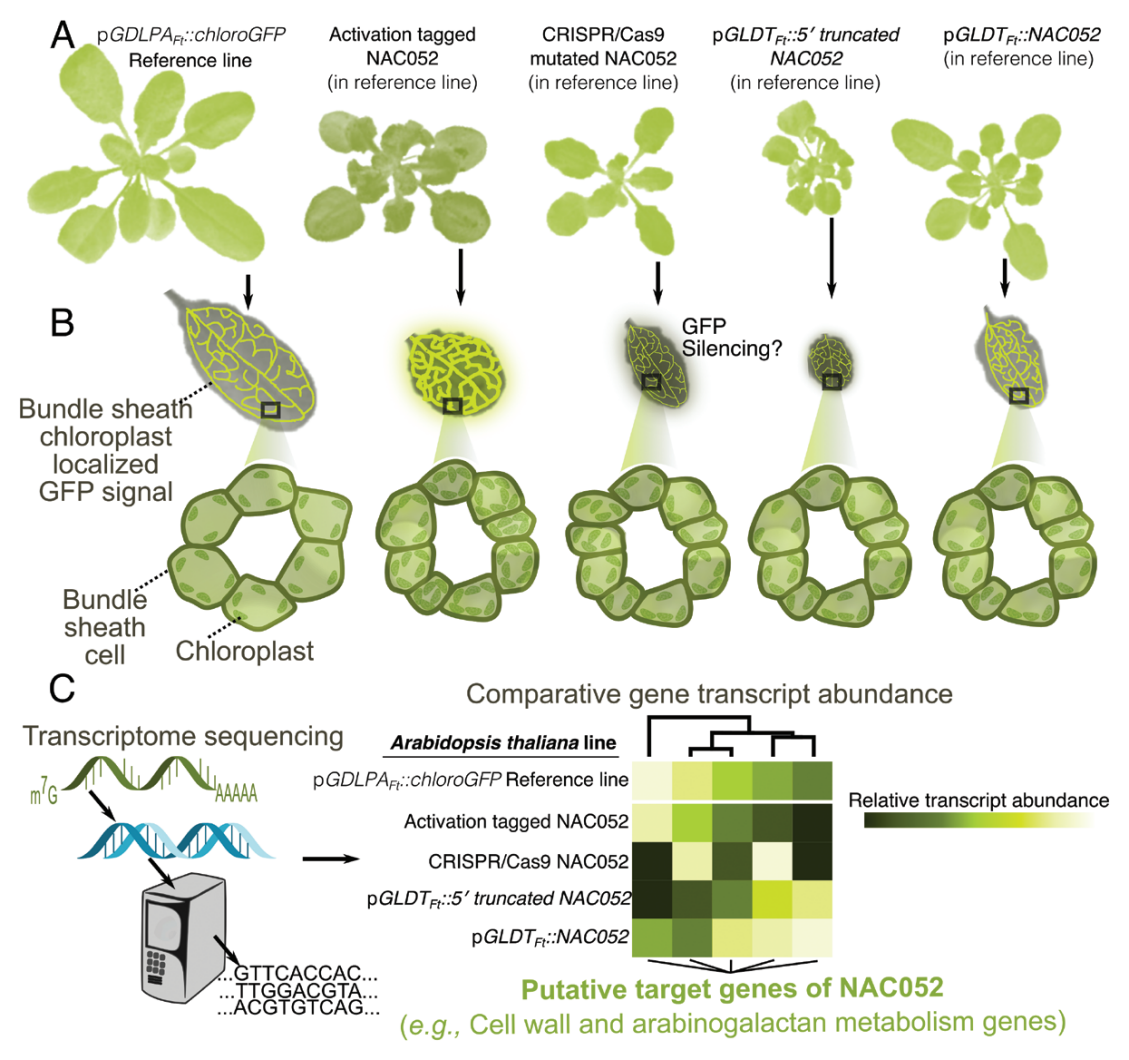

The insertion event resulted in a 5′-truncated transcript variant of NAC052 leading to a partial deletion of its DNA-binding domain. The mutation led to changes in GFP fluorescence as well as changes in BS anatomy and leaf and whole-plant morphology, such as a greater number of BS cells and chloroplasts as compared with the reference line. The JMJ14–NAC052 module is involved in post-transcriptional gene silencing by acting as a H3K4 demethylase which promotes transgene transcription by preventing DNA methylation (Butel *et al.*, 2017). Furthermore, the activation tagging mutation event was reconstructed by expressing pGLDT_Ft_::5′-truncatedNAC052 in the pGLDPA_Ft_::RbcS.TP-sGFP reference line. This recapitulated the previous chlorotic and wrinkled leaf edge phenotypes and caused a greater accumulation of BS cells as compared with the reference line. Further validation of the involvement of NAC052 in leaf development was confirmed by expressing the full reading frame of the NAC052 transcript under the control of the GLDT_Ft_ promoter in the pGLDPA_Ft_::RbcS.TP-sGFP reference background (pGLDT_Ft_::NAC052). The lines showed enhanced GFP signal intensity and more BS cells. As an additional line of evidence of NAC052 function, the endogenous NAC052 was mutated with clustered regularly interspaced short palindromic repeats (CRISPR)/CRISPR-associated protein 9 (Cas9). The CRISPR/Cas9 mutant line was small and had chlorotic leaf edges, but it did not show the wrinkled leaf edges. However, in contrast to the activation-tagged mutant, the GFP signal intensity was decreased in the CRISPR/Cas9 mutant as compared with the reference line.

To assess the genetic impact of ectopic NAC052 expression, mRNA sequencing of the reference and all the transgenic lines was undertaken to identify its downstream gene regulatory targets. Comparative transcriptomics of the lines showed differential transcript abundance of genes involved in leaf cell wall organization and arabinogalactan metabolism, which are mediators between the cell wall, the plasma membrane, and the cytoplasm. In summary, van Rooijen *et al.* (2019) associate NAC052 with leaf developmental patterns that alter anatomy specifically related to Kranz-like features. This opens up exploration into the role of other *trans*-factors that may have arisen from existing regulatory networks to transition from an ancestral C_3_ state to a derived C_4_ photosynthetic state.

## Future perspectives

The study from van Rooijen *et al.* (2019) is an advancement in our current understanding of BS anatomical regulation and furthers investigation into the role of post-transcriptional gene silencing in leaf development. Incorporation of bisulfite sequencing and methylome data sets might uncover underlying epigenetic patterns affecting BS anatomy and function across C_3_ and C_4_ species.

Here, van Rooijen *et al.* found that NAC052 had a transcriptionally repressive role in C_3_*A. thaliana*, which caused a boost in BS number and chloroplast content when ectopically expressed. Extension of their methodology to a C_4_ species would allow association of NAC052 with traits of Kranz anatomy. Coupled with putative regulators of cell and plastid division genes, misexpression of NAC052 might shed insights into a mechanistic understanding of gene-regulatory networks that enhance the photosynthetic capacity of the BS. It would be particularly interesting to see if this could lead to trait stacking for efforts to engineer C_4_ photosynthesis in C_3_ crops, such as increased vein density or metabolic flux between M and BS cells from more plasmodesmata connections.

To sum up, van Rooijen *et al.* (2019) highlight how high-throughput phenotyping of transgenic activation-tagged lines can uncover novel gene-regulatory networks. This expands knowledge on how to manipulate the role and structure of the BS in C_3_ species. Use of forward genetics like this seems to be a promising approach to unravel the complexity of Kranz anatomy. Hopefully this will lead to further reports that identify genetic determinants underpinning the regulation of Kranz traits in C_4_ photosynthesis.
